# Formation of Ultrafine-Grained Dual-Phase Microstructure by Warm Deformation of Austenite in High-Strength Steel

**DOI:** 10.3390/ma18061341

**Published:** 2025-03-18

**Authors:** Wen Shu, Yingqi Fan, Rengeng Li, Qing Liu, Qingquan Lai

**Affiliations:** 1 Key Laboratory for Light-weight Materials, Nanjing Tech University, Nanjing 211816, China; 202261203266@njtech.edu.cn (W.S.); 2222205128@stmail.ujs.edu.cn (Y.F.); liuqing@njtech.edu.cn (Q.L.); 2 Materials Academy, Jiangsu Industrial Technology Research Institute, Suzhou 215131, China

**Keywords:** deformation-induced ferrite transformation, ultrafine-grained steels, microstructure, kinetics, thermomechanical processing

## Abstract

Thermomechanical processing by applying deformation-induced ferrite transformation (DIFT) is an effective method of producing ultrafine-grained (UFG) ferritic steels, which usually present high yield strength but low strain hardening. In this study, we explored the concept of DIFT in the processing of UFG dual-phase (DP) steel, in order to improve its strain hardening capability and thus its ductility. The processing temperature was reduced to enhance the dislocation storage in austenite. It was found that the warm deformation of austenite induced a dramatic occurrence of DIFT, resulting in the formation of UFG-DP microstructures along the whole thickness of the specimen. In the UFG-DP microstructure, the average ferrite grain size was 1.2 μm and the ferrite volume fraction was 44 vol.%. The observation of twinned martensite suggests the occurrence of carbon partitioning during the DIFT process. The UFG-DP microstructure exhibited a good combination of strength and ductility, which was enabled by the synergy of the ultrafine ferrite grains and the efficient composite effect. The outcome of this study provides a novel pathway to develop advanced hot-rolled steels with a UFG-DP microstructure and which are associated with the advantages of their readiness to be scaled up and low costs.

## 1. Introduction

High-strength steels play a critical role in numerous modern industries, including the automotive and infrastructure industries. High-strength steel grades could be produced traditionally by post-rolling heat treatments, such as quench and tempering, for the desired mechanical properties [1]. Significant advances have been made in the field of thermomechanical processing (TMCP), which is a metallurgical technology that integrates plastic forming and heat treatment into a single process. TMCP has shown a remarkable capability in optimizing the microstructural features and in improving the mechanical properties of the steel products [2,3,4].

For hot-rolled steels, by controlling the rolling temperature, the rolling strain and the strain rate, the dislocation substructure can be sufficiently introduced and stored in the austenite grains, and the deformed austenite presents different phase transformation behavior when compared to the annealed counterpart. Generally, the ferrite transformation is accelerated by the austenite deformation [5], but the martensitic transformation can be suppressed by the so-called mechanical stabilization effect [6,7]. The acceleration of ferrite transformation from the deformed austenite could be reflected by its occurrence above the A_r3_ or even the A_e3_ temperature [5,8,9]. During the hot rolling, the concurrence of ferrite transformation and ferrite recrystallization results in the formation of ultrafine-grained (UFG) ferrite, with the grain size reduced to 1–3 μm [10,11,12,13]. The deformation-induced ferrite transformation (DIFT) provides a promising pathway to produce ultrafine-grained steels, requiring a much smaller accumulated plastic strain than the techniques of severe plastic deformation [14,15,16].

Extensive investigations have been made to clarify the micromechanism of deformation-induced ferrite transformation [9,10,17,18,19,20,21]. From the thermodynamic perspective, additional driving force is provided by the stored energy in austenite or by the applied stress that works to accommodate the transformation strains [15,22,23]. From the kinetics perspective, the plastic strain induces the dislocation substructure that provides intensive nucleation sites for ferrite and also enhances the diffusivity of the alloying elements [24,25]. Both perspectives suggest that the ferrite transformation and the ferrite grain refinement are highly dependent on the characteristics of the dislocation substructure in the austenite grains. Microalloying elements (e.g., Nb and Ti) were found to enhance the efficiency of the ferrite grain refinement, which was attributed to the enhanced dislocation storage in austenite via the strong dislocation pinning by the nano-sized carbide [26,27,28]. However, the previous methods generally induced the UFG microstructure in the near-surface region [10,11,27], and it remains a challenge to generate the UFG microstructure along the whole section thickness of the sheet products. Decreasing the deformation temperature could provide a possibility for this purpose, if one considers the effect of deformation temperature on the kinetics of recovery and recrystallization.

The benefits of forming UFG ferrite by exploring the deformation-induced ferrite transformation have been widely reported [14,15,29], including the increase in strength and the decrease in ductile-to-brittle transition temperature. However, the UFG ferrite usually presents a discontinuous yielding behavior, associated with a negligible strain hardening capability [10,30,31]. The strain hardening rate and thus the strength–ductility balance could be improved by introducing the reinforcing martensite phase, i.e., the generation of UFG dual-phase (DP) microstructures [20,32]. However, the application of the DIFT to process UFG-DP steels is still open for research, and the guidelines for optimizing the UFG-DP microstructure are not yet established. In previous studies, the UFG-DP steels were mainly produced by the techniques of severe plastic deformation and flash heat treatment [33,34], the implementation of which to real industrial lines remains challenging.

In this study, a steel grade with a high hardenability was used, which allowed a systematic investigation of the austenite deformation at a relatively low temperature (650 °C). The warm deformation of austenite should presumably promote the occurrence of DIFT and also the generation of the UFG-DP microstructure as a bulk material. The microstructure evolution with the increasing strain of the warm deformation of austenite was studied, and the mechanical properties of the UFG-DP steels were assessed by the uniaxial tensile tests. The materials and experimental methods used in this study are detailed in Section 2. The results are presented in Section 3, which is followed by the discussion in Section 4 and the conclusions in Section 5.

## 2. Materials and Methods

The steel used in this study was in the form of a hot-rolled plate (13 mm in thickness), and the chemical composition was Fe-0.16C-0.16Si-1.29Mn-0.4Cr (wt.%). Dilatometry tests were performed on the as-received materials with the DIL805A/D dilatometer (TA Instruments, New Castle, DE, USA). The specimens of 3 mm in diameter and 6 mm in length were heated to 900 °C with a heating rate of 15 °C/s for austenitization. Subsequently, either direct quenching to room temperature (RT) or isothermal tests at elevated temperatures were performed to measure the transformation.

The thermomechanical processing was performed by compression on the Thermecmastor simulator (Fuji Electronic Ind. Co., Shizuoka, Japan). Cylindrical specimens with a diameter of 8 mm and a length of 12 mm were machined. The specimens were heated and held at 900 °C for 20 min and then quenched to the designated temperature, based on the results of the dilatometric measurements. After quenching, the specimens were held for 5 s. Thereafter, compression was carried out with a reduction of 20%, 30%, 40% and 60%, respectively, with a strain rate of 1/s, and was finished by rapid cooling to RT.

Microstructure characterizations were made at the center of the compressed specimens after cutting, grinding and polishing. To reveal the microstructures for SEM observations, the samples were etched with 2% Nital. For the EBSD characterization, the samples were polished with the colloidal silica. A step size of 70 nm was used in the EBSD scanning. Thin-film specimens for the transmission electron microscope (TEM) observations were ground to a thickness of 50 μm, and electrolytical polishing was performed with an 8% ethanol perchlorate solution at −20 °C. The observations were conducted with the TEM operated at 200 kV.

Dog-bone-shaped tensile specimens with a length of 4.2 mm, a thickness of 0.8 mm and a width of 1 mm of the gauge section were cut from the compressed specimens. Uniaxial tensile tests were conducted at room temperature with a strain rate of 1 × 10^−3^/s on an Instron 5982 mechanical testing machine, which was equipped with the optical extensometer to measure the strain in the gauge section. The images of the tensile specimen and the setup of the tensile testing are provided in the Supplementary Materials. Three tests were repeated for each microstructural condition.

## 3. Results

**Figure 1:**
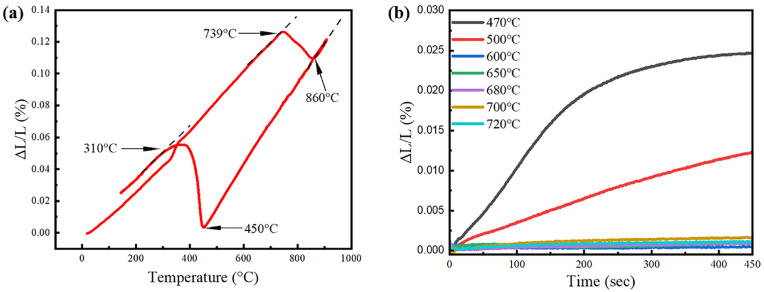
The phase transformation of the steel grade under investigation. (**a**) is the dimensional change during the heating and quenching cycle; and (**b**) is the kinetics of isothermal transformation.

Figure 1 shows the results of the phase transformations in the steel grade under investigation. The A_e1_ and A_e3_ temperatures are 739 °C and 860 °C, respectively. Upon quenching, the martensitic transformation occurs at 450 °C. While being held above the martensite start temperature, the bainitic transformation occurs at the temperatures of 470 °C and 500 °C. However, the well-annealed austenite is stable at the temperature ranging from 600 °C to 720 °C, without the occurrence of isothermal transformation.

**Figure 2:**
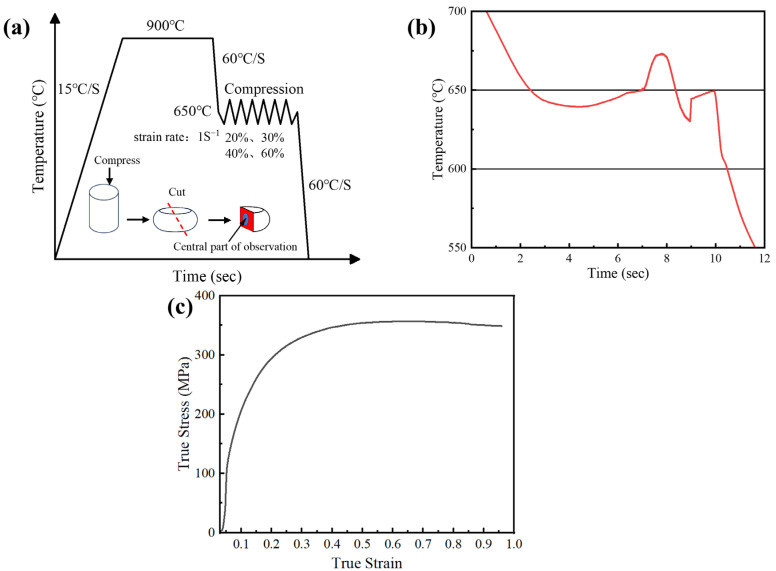
Compression tests using the thermomechanical simulator. (**a**) The schematic illustration of the compression test; (**b**) the temperature variation during the holding and compression; and (**c**) the rvolution of the flow stress during the compression.

In this study, 650 °C is selected as the compression temperature for TMCP, where the high stability of the undercooled austenite allows for a detailed investigation of the effect of austenite deformation. Figure 2 shows the details of the compression tests. Some temperature variation occurs during the holding and compression operations, but the temperature remains outside of the range for bainitic transformation, as shown in Figure 1b. Significant strain hardening is observed during the compression, and the flow stress is saturated at the true strain of ~0.65.

**Figure 3:**
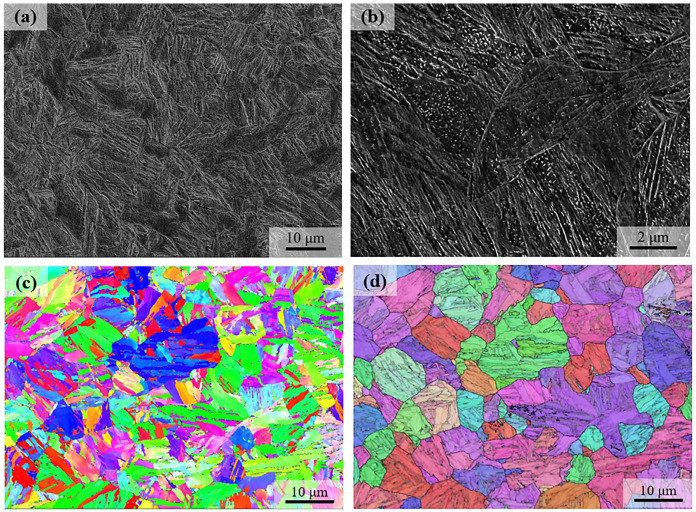
The microstructure after quenching. (**a**,**b**) are SEM micrographs; (**c**) is the Inverse Pole Figure (IPF) of the body-centered cubic (BCC) phase; and (**d**) is the reconstruction of the prior austenite grains from the EBSD data. The different color in (**d**) indicates different orientation of the prior austenite grains.

Figure 3 shows the microstructure of the as-quenched martensite without hot compression, which is of a typical lath morphology. Carbide particles can be observed, which are presumably a result of the autotempering, in that the martensite start temperature is well above RT. The crystallographic features of the lath martensite are shown by the Inverse Pole Figure (IPF) in Figure 3c. The austenite reconstruction is accomplished with a high quality, which shows the equiaxed austenite with an average grain size of 9 μm.

**Figure 4:**
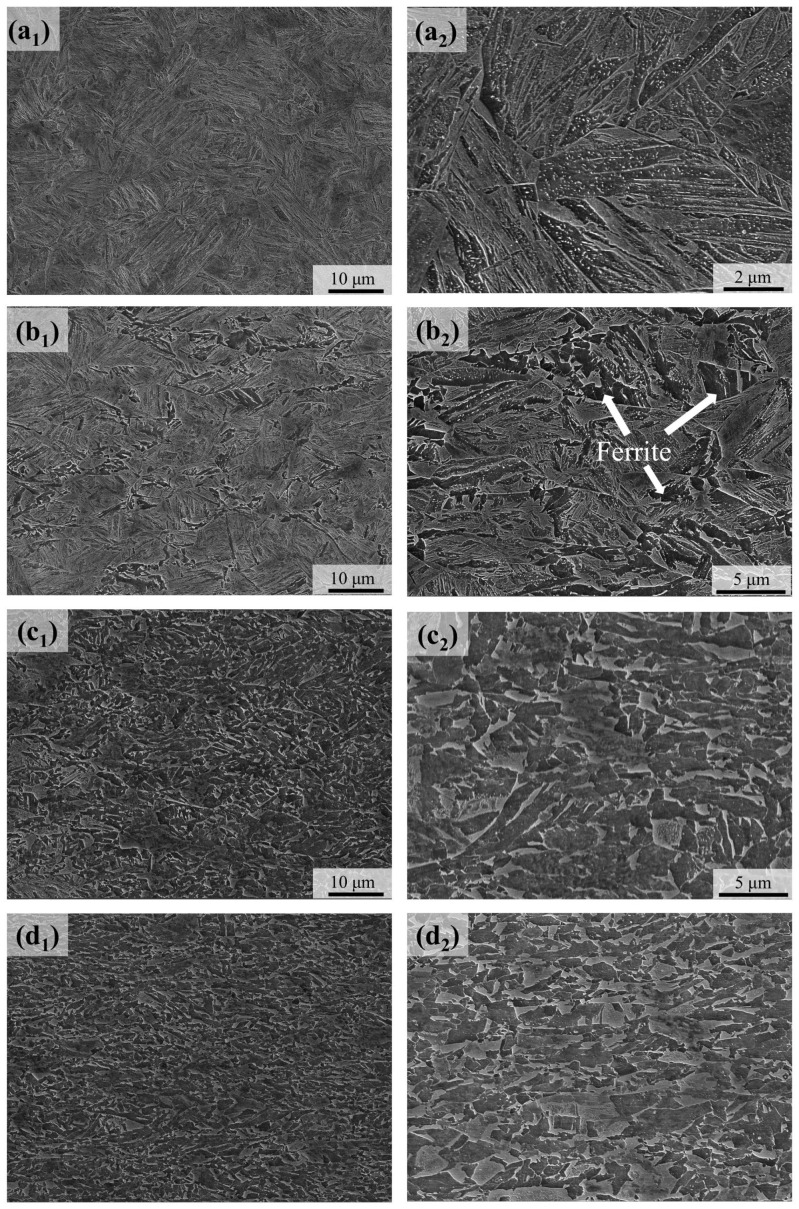
Microstructures after different levels of compression. (**a_1_**–**d_2_**) are the SEM micrographs of the microstructures after the compression reduction of 20%, 30%, 40% and 60%, respectively.

Figure 4 shows the microstructure evolution after different levels of hot compression. For the compression by 20% reduction, the microstructure remains of the fully lath morphology. When the compression reduction was increased to 30%, a small amount of ferrite could be observed, mainly at the locations of the prior austenite grain boundaries. For the compression reduction of 40%, a large amount of ferrite was formed, associated with a complex mixture with martensite. It is clear that the compression of 60% resulted in well-defined ultrafine-grained dual-phase steels, associated with the average size of the ferrite grains being 1.2 μm and the ferrite volume fraction being 44 vol.%. On the contrary to the absence of isothermal transformation in the well-annealed austenite, the deformation-induced ferrite transformation, and thus the formation of the UFG-DP microstructure, was mainly accomplished within a fast compression step (strain rate of 1/s) and subsequent cooling.

**Figure 5:**
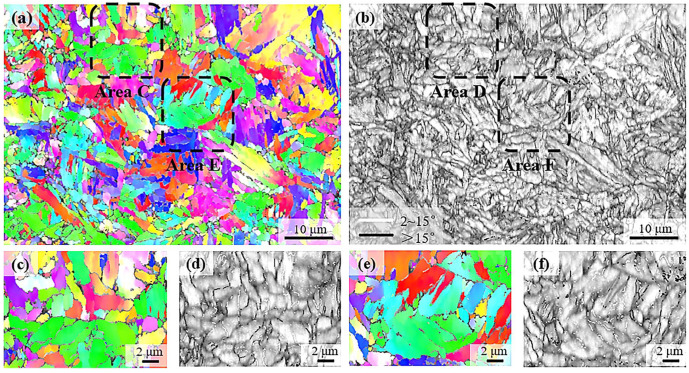
EBSD characterization of the microstructure after compression by 30%. (**a**) is the IPF of the BCC phase; (**b**) is the distribution of high-angle grain boundaries (>15°) and low-angle boundaries (<2°) superimposed with the band contrast. (**c**–**f**) are the magnified micrographs of the selected areas as indicated in (**a**) and (**b**).

Figure 5 reveals the microstructure of the sample after compression by 30%. This microstructure presents a more complex morphology than the as-quenched one, as shown in Figure 3. The microstructural heterogeneity is obvious, including both the typical martensite laths with maximum length of ~30 μm and the granular grains of a few micrometers in size. Such microstructural heterogeneity might reflect the heterogeneity of the austenite grain size by this level of compression.

**Figure 6:**
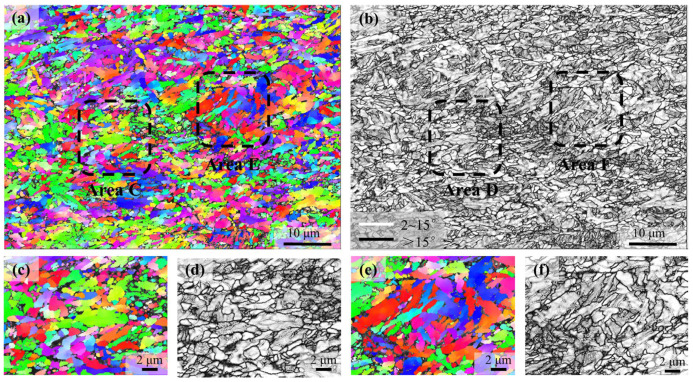
EBSD characterization of the microstructure after compression by 60%. (**a**) is the IPF of the BCC phase; (**b**) is the distribution of high-angle grain boundaries and low-angle boundaries superimposed with the band contrast. (**c**–**f**) are the magnified micrographs of the selected areas as indicated in (**a**) and (**b**).

Figure 6 shows the EBSD results of the microstructure after compression by 60%. Consistent with the SEM observations, this microstructure mainly involves the ultrafine grains with irregular grain shape; although, with a large amount of martensite islands, as shown in the SEM, the lath morphology is not obvious in the microstructure revealed by EBSD. In addition, the band contrast could not be used to distinguish the ferrite and martensite in this case, which is primarily attributed to the high dislocation density in ferrite.

**Figure 7:**
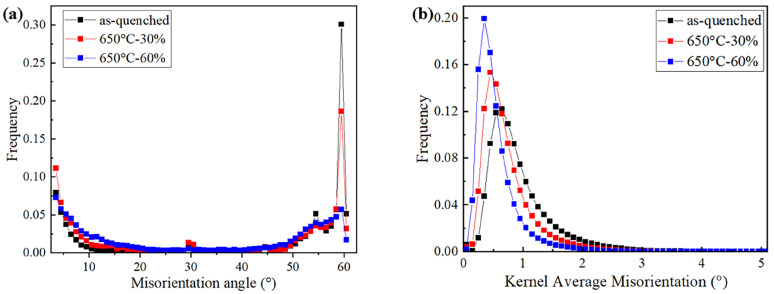
Statistics of the crystallographic information obtained by EBSD. (**a**) The distribution of the misorientation angles; and (**b**) the distribution of the KAM values.

The difference in the microstructure can be further studied by statistical analysis of the crystallographic information. As shown in Figure 7a, the as-quenched sample presents the peaks of the boundary misorientations. The peak at 54° is characteristic of the Nishiyama–Wassermann orientation relation and the peak close to 60° is characteristic of the Kurdjumov–Sachs relation [35]. However, the increase in compression reduction changes the distribution of the misorientation angle, especially for the diminishing peak close to 60°, which indicates a different scenario of microstructure generation. In addition, as shown in Figure 7b, it is found that, with an increase in compression reduction, the KAM value decreases instead. Noticeably, the sample after compression by 60% involves a high proportion of low KAM values, which is suggested to correspond to the large amount of ferrite phases.

**Figure 8:**
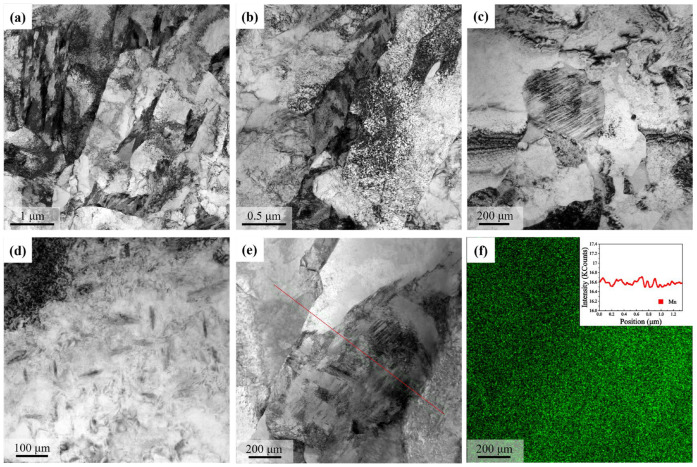
TEM observations of the microstructure after 60% hot compression. (**a**–**e**) are bright-field micrographs; and (**f**) is the distribution of Mn as measured by TEM-EDX. The line analysis in (**f**) is made along the red line marked in (**e**).

The microstructure after 60% compression was characterized in detail by TEM. As shown in Figure 8, the ultrafine-grained microstructure was clearly formed, including both the ferrite and the martensite phases (Figure 8a). The dislocation substructure within the ferrite was heterogenous, and a high density of tangled dislocations were formed in the ferrite region adjacent to the martensite island (Figure 8b). The nanotwins in the martensite islands were frequently observed (Figure 8c), which indicated a medium-to-high carbon content and was presumably a result of the carbon partitioning during the austenite-to-ferrite transformation. Nano-sized carbide particles with a needle shape could be found in the ferrite (Figure 8d). The TEM-EDX measurement was made in an area with both ferrite and martensite, and the distribution of manganese was found to be homogeneous, according to both the mapping and line analysis in Figure 8f, which suggested the absence of Mn partitioning during the austenite-to-ferrite transformation.

**Figure 9:**
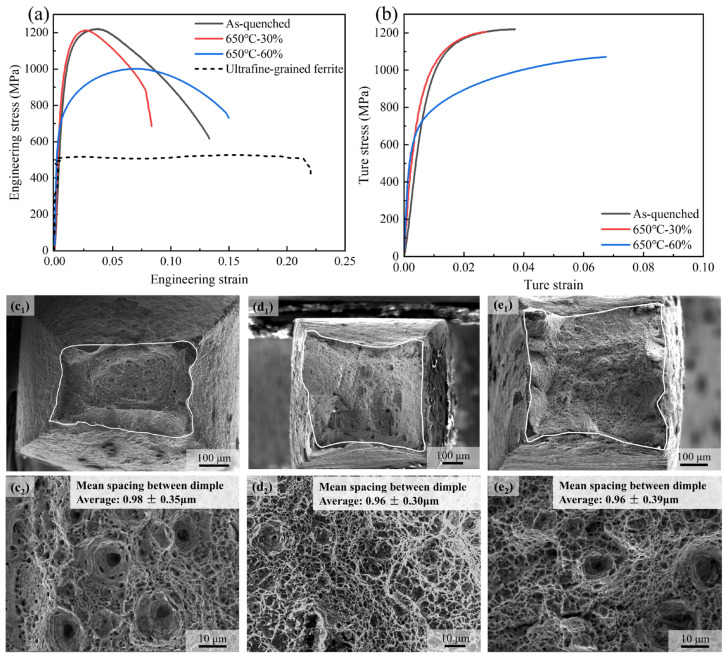
Tensile response of the materials. (**a**) The engineering stress–strain curves; (**b**) the true stress–strain curves; and (**c1**–**e2**) the fracture surface observations of the as-quenched 650 °C—30% and 650 °C—60% samples, respectively. The data of the stress–strain curves of UFG ferrite in (**a**) are taken from ref. [10].

The mechanical consequences of the austenite deformation are assessed by uniaxial tensile tests. As shown in Figure 9, the as-quenched martensite, even with autotempering, still presents a high tensile strength of 1250 MPa and a large post-necking deformation. For the sample after compression by 30%, the strength is slightly reduced due to the formation of a small amount of ferrite, while the area reduction in the tensile specimen is significantly reduced when compared with the as-quenched martensite. For the sample after compression by 60%, the yield strength is significantly reduced due to the much larger amount of ferrite phases, but the salient strain hardening capacity in the UFG-DP microstructure results in a 1GPa tensile strength and a much higher uniform elongation (0.07), which indicates the potential benefits of the forming process, such as bending. The UFG-DP microstructure exhibits a well-rounded stress–strain curve, which is contrary to the discontinuous yielding of the UFG ferrite. The area reduction in the hot-compressed samples is lower than the as-quenched one, but the mean spacing between the dimple centers are similar.

## 4. Discussion

Austenite deformation invokes significant impacts on the subsequent phase transformations during cooling or isothermal holding, constituting a flexible and effective approach of TMCP to the processing of novel steels with an improved mechanical performance. The austenite deformation generally accelerates the austenite-to-ferrite transformation, and the deformation-induced ferrite transformation could occur at the temperature above the A_r3_ [5]. Furthermore, when the strain accumulation is sufficient, the ferrite transformation could even occur above the A_e3_, which is evidenced by the experimental measurements [9]. The results of this study provide a particular example of the acceleration of ferrite transformation in an undercooled steel with a high hardenability. As shown in Figure 1b, the well-annealed austenite of the previous steel grade is kinetically stable between 600 °C and 720 °C, suggesting a high hardenability to postpone ferrite transformation. However, after the warm deformation at 650 °C, a large amount of ferrite is formed within a very short duration (Figure 4), instead of being formed during the isothermal holding, as reported in [24]. Here, in this case, the driving force for ferrite transformation is not an issue, since the austenite is highly undercooled, and the activation of ferrite transformation should be attributed to the kinetic effects of the austenite deformation [15].

There are two possible contributions of the austenite deformation to the kinetics of subsequent ferrite transformation, including the modification of austenite grain shape and the generation of dislocation substructures within the austenite grains. On one hand, the modification of austenite grain shape increases the area of the grain boundaries, thus providing more potential sites for ferrite nucleation. However, this point is not supported by the results in Figure 1, in that the grain boundaries in the well-annealed austenite are not inducing ferrite transformation at the present temperature. On the other hand, the plastic deformation of austenite could generate a set of dislocation substructures, the characteristics of which are influenced by the deformation temperature and strain. According to the systematic observations on the model alloy Ni-30Fe [36], which is of comparable stacking fault energy with the austenite in pure iron and low-alloyed steels, dislocation cells are formed at a high deformation temperature (e.g., 800 °C) and the cell boundaries are sharper and associated with a larger misorientation when the strain is increased; while at a lower deformation temperature (down to 530 °C), microbands or geometrically necessary boundaries [37] with a larger misorientation are formed, accompanying the dislocation cells, and the microbands become dominant at large strains. The deformation temperature and strain in this study (650 °C, 60%) suggest the extensive formation of microbands in the austenite. It is suggested that a critical subgrain misorientation is required to activate the potential nucleation sites, and the critical subgrain misorientation is more likely to be attained first in the vicinity of the austenite grain boundaries [24]. This is consistent with the observations in this study and the previous literature results [12,38,39], i.e., the deformation-induced ferrite transformation occurs first at austenite grain boundaries beyond a critical strain. When the strain is increased, the misorientation in the cells and microbands is increased, reaching the critical condition to activate the intragranular nucleation of ferrite. The warm deformation, when compared with the TMCP at higher temperatures, should be more efficient in dislocation storage, and thus the increase in boundary misorientation occurs, leading to the activation of substantial ferrite nucleation sites. This is reflected by the ultrafine ferrite grain size of 1.2 μm of the whole bulk specimen with a thickness of 3.2 mm. As a comparison, the achievement of the UFG microstructure is generally limited to the near-surface region of the hot-rolled sheet product [10,11,27]. Notice that the achievement of the UFG ferrite could also be facilitated by the dispersion of austenite islands, the pinning effect of which helps in maintaining the ultrafine grain size.

The detailed TEM observations in Figure 8 provide critical information to understand the DIFT, which remains an issue under debate [15,16]. Firstly, the martensite islands are observed to involve the formation of nanotwins, which is the typical feature of martensite with a medium-to-high carbon content when compared with the low-carbon lath martensite with a high dislocation density [40]. Secondly, the manganese is distributed uniformly within the microstructure. Thirdly, the carbide particles with a size below 20 nm are found within ferrite, which indicates the supersaturated state of the deformation-induced ferrite [16]. Therefore, during the DIFT, the carbon element is suggested to be partitioned to the austenite islands, and the enrichment of carbon in austenite leads to the formation of twinned martensite after quenching. However, the partitioning of substitutional elements is not involved, as there is not enough time for this to take place. The freshly formed ferrite should be initially supersaturated with carbon, which is rejected to form the carbide particles during cooling.

The generation of the UFG-DP microstructure results in a good combination of strength and ductility. According to the data in [31], the deformation-induced ferrite with a grain size of 1 μm presents a yield strength of 500 MPa, with discontinuous yielding and negligible strain hardening, which is actually not the most favored for engineering applications. As a comparison, the UFG-DP microstructure in this study exhibits a yield strength of 640 MPa, associated with continuous yielding, and the salient strain hardening allows for a true tensile strength of 1100 MPa and a true uniform elongation of 0.07. This suggests that the martensite phase in the UFG-DP microstructure is still of sufficient strength in contrast to the UFG ferrite, which is essential to induce the composite effect for the salient strain hardening capability [41,42]. The high strength of the martensite phase is primarily enabled by the increased carbon content due to the carbon partitioning during the deformation-induced ferrite transformation. Notice that the tensile properties of the hot-rolled UFG-DP in this study are comparable to the cold-rolled counterparts processed by more complicated methods, such as severe plastic deformation and flash heat treatment [33,43,44]. It is believed that the deformation-induced ferrite transformation would allow for the development of hot-rolled or hot-worked steels with UFG-DP microstructures [45,46]. The processing route and parameters in this study with the thermomechanical simulator should have the potential to be transferred to real industrial practice.

## 5. Conclusions

In this study, the warm deformation of austenite was performed in a high-strength steel. During the conventional thermal cycle, the well-annealed austenite presented a high stability at the temperature of 650 °C, and the isothermal transformation was absent. On the contrary, the warm deformation of austenite at this temperature induced a dramatic formation of ferrite, evidencing the occurrence of deformation-induced ferrite transformation. A compression reduction of over 30% was required to induce the formation of ferrite, and a compression reduction of up to 60% resulted in the formation of ultrafine-grained dual-phase steels with a ferrite grain size of 1.2 μm and a ferrite volume fraction of 44%. The TEM observations of the twinned martensite suggested carbon partitioning during the deformation-induced ferrite transformation. The ultrafine-grained dual-phase microstructure presented a good combination of strength and ductility, which was enabled by the synergy of the ultrafine ferrite grains and the efficient composite effect. The simple thermomechanical processing by the warm deformation of austenite provided a potential pathway for developing hot-rolled steel products with UFG-DP microstructures, which should have the advantages of their readiness to be scaled up and low costs.

## Data Availability

The original contributions presented in this study are included in the article/Supplementary Materials. Further inquiries can be directed to the corresponding authors.

## Supplementary Materials

The following supporting information can be downloaded at: https://www.mdpi.com/article/10.3390/ma18061341/s1, Figure S1: Experimental setup of the uniaxial tensile test. (a) Contactless strain measurement based on digital image correlation (DIC) technique; (b) Tensile specimen with surface speckle pattern; (c) DIC images showing the strain distribution along tensile direction.
