# Supplementary material for: Formation of Ultrafine-Grained Dual-Phase Microstructure by Warm Deformation of Austenite in High-Strength Steel
Source: Materials (Basel). 2025 Mar 18;18(6):1341. doi: 10.3390/ma18061341 (PMC11943922; doi:10.3390/ma18061341)
Supplement: Supplementary file 1 [file materials-18-01341-s001.zip › materials-3502414-supplementary.pdf]

Supplementary Materials for

Formation of ultrafine-grained dual-phase microstructure by warm deformation of austenite in a high-strength steel

Wen shu[1+], Yingqi Fan[1+], Rengeng Li[1*], Qing Liu[1,2], Qingquan Lai[1,2*]

1 Key Laboratory for Light-weight Materials, Nanjing Tech University, Nanjing, People's Republic of China;

2 Materials Academy, JITRI, Suzhou, People's Republic of China.

+Contributing equally

*Corresponding authors: lai@njtech.edu.cn; lirengeng@njtech.edu.cn.

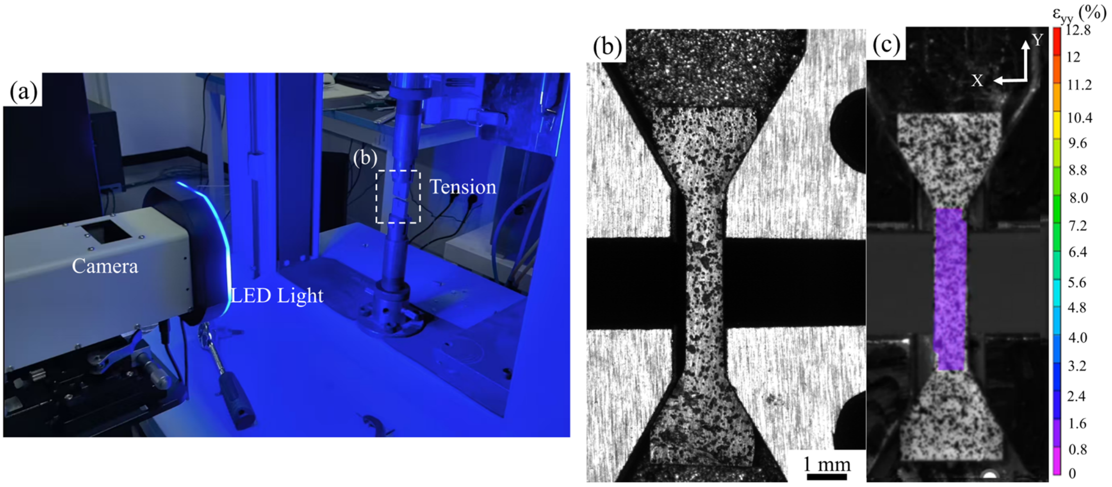

Figure S1: Experimental setup of the uniaxial tensile test. (a) Contactless strain measurement based on digital image correlation (DIC) technique; (b) Tensile specimen with surface speckle pattern; (c) DIC images showing the strain distribution along tensile direction.
